# Long-term conservative treatment outcomes for midshaft clavicle fractures: a 10-to-30-year follow-up

**DOI:** 10.1186/s13018-023-04450-9

**Published:** 2023-12-11

**Authors:** Yuki Matsubara, Yoshihiro Nakamura, Yoshiaki Sasashige, Shin Yokoya, Nobuo Adachi

**Affiliations:** 1 Department of Orthopaedic Surgery, Chugoku Rosai Hospital, 1-5-1, Tagaya, Hiro, Kure, Hiroshima 737-0193 Japan; 2 Department of Orthopaedic Surgery, Hiroshima Citizens Hospital, 7-33 Motomachi, Naka-ku, Hiroshima 730-8518 Japan; 3 Department of Orthopaedic Surgery, Hiroshima University, 1-2-3 Kasumi, Minami-ku, Hiroshima 734-8551 Japan

**Keywords:** Midshaft clavicle fracture, Conservative treatment, Long–term outcomes, Figure-of-eight bandage

## Abstract

**Background:**

Few reports exist on the long-term outcomes of midshaft clavicle fracture conservative treatments. Therefore, this study investigated the long-term outcome of this treatment in patients with midshaft clavicle fractures.

**Methods:**

Patients were treated conservatively for midshaft clavicle fractures with a figure-of-eight bandage between 10 and 30 years ago. Subsequently, a telephone survey was used to follow-up these patients, and 38 were successfully evaluated. The mean term after trauma was 17.0 years. Afterward, the American Shoulder and Elbow Surgeons Shoulder (ASES) score and Shoulder pain and disability index (SPADI) on the affected and unaffected sides were calculated based on the filled questionnaires. We defined patients whose ASES and SPADI on the affected side were worse than the unaffected side as the symptomatic group. Furthermore, plain radiographs measured proportional changes in clavicular length and displacement.

**Results:**

The ASES scores of the affected side were significantly lower than those of the unaffected side, and the SPADI of the affected side was significantly higher than that of the unaffected side. Furthermore, the symptomatic group's proportional changes in clavicular length and displacement were significantly larger than the asymptomatic group.

**Conclusion:**

The affected shoulder side was more symptomatic than the unaffected side 10 to 30 years after the trauma when midshaft clavicle fractures were treated conservatively. Moreover, several patients became symptomatic for fractures with a larger proportional change in clavicular length or displacement.

## Background

Clavicle fractures are common and comprise 2.6–10% of all bone fracture cases [1], with many of those occurring in young adults. While over one-third of clavicle fractures in males occur between 13 and 20 years old, 20% occur in females at the same age [2]. A clavicle fracture can be classified as a proximal, midshaft, or distal fracture. Although midshaft clavicle fractures account for 69–82% of clavicle fractures [2, 3] and are treated conservatively and surgically, some reports said that the rate of nonunion after midshaft clavicle fracture treated conservatively was low [3–5]. Other studies have reported that surgical treatment reduced the incidence of nonunion compared with conservative treatments [6–9]. Several other reports also exist on the short-term outcome of conservative treatments for midshaft clavicle fractures [6–12]. Pathak et al. reported that compared with surgical treatments, conservative treatments using a figure-of-eight bandage showed no significant difference in the functional outcome [9]. Other reports suggesting that conservative treatments provided less short-term satisfaction to patients than surgical treatments after trauma have also been observed [8, 10]. It was reported that shortening of the clavicle does not reduce shoulder function short or middle term after trauma [12–14]. Furthermore, although scapulohumeral kinematics at the shoulder of the affected side differed from those of the unaffected side, these changes did not result in any clinical change [15]. However, only a few reports on the long-term follow-up outcomes in patients with midshaft clavicle fractures exist [16, 17].

The purpose of our study was to survey the long-term outcome of conservative treatments for midshaft clavicle fractures. It was hypothesized that clavicular shortening due to clavicle fractures resulted in poor clinical outcomes long term after conservative treatments for midshaft clavicle fractures.

## Materials and methods

**Fig. 1:**
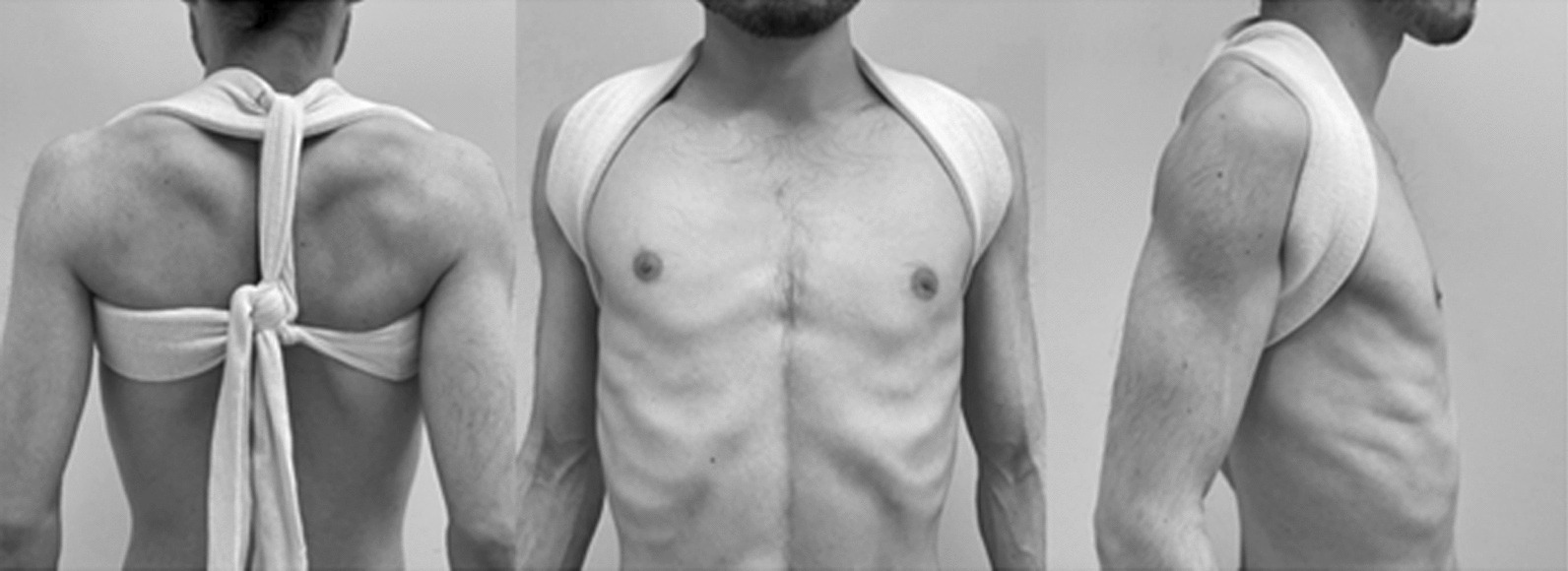
Rigidly fastened figure-of-eight bandage

The patients and their family were informed that data from the research would be submitted for publication, and gave their consent. The institutional review board approved this retrospective study. All patients with midshaft clavicle fractures were conservatively treated for between 1990 and 2010. The conservative treatment was performed for 222 patients with a figure-of-eight bandage between 6 and 9 weeks, which orthopedic surgeons rigidly fastened twice or three times a week (Fig. 1). Plain radiographs were taken until bone union. Four patients underwent surgery secondarily for delayed union. Then, 218 patients who continued conservative treatment were telephoned in 2020, and 40 patients answered the telephone. Those that responded were taken as the study participants and were scheduled for a questionnaire survey. However, two patients were excluded because while one developed clavicle fractures on both sides afterward; the other had developed hemiplegia due to cerebral hemorrhage. Therefore, the total participants were 38 patients (31 males and 7 females). The mean age during the survey was 60.4 ± 16.0 (24–86) years old, the age of period of trauma was 43.4 ± 16.5 years old, and the mean term after trauma was 17.0 ± 4.6 years.

We asked 19 questions during the phone survey concerning both shoulder sides' degree of pain and difficulties experienced in daily activities on a scale of 0–10. In detail, questions were asked about the present degree of usual pain, their worst pain, pain felt while lying on their affected or unaffected side, pain felt while reaching for something on a high shelf, pain felt while placing their hands behind their neck, and pain felt while pushing something. Additionally, questions were asked about difficulties experienced in putting on undershirt or jumpers, shirts that buttons down at the front and pants, lying on their affected or unaffected side, washing their back and heir, touching on their hip, removing something from back pockets, placing an object on a high shelf, throwing balls, carrying a heavy object of 10 pounds, and doing work, hobbies, and house chores.

This study used the American Shoulder and Elbow Surgeons (ASES) score and the Shoulder Pain and Disability Index (SPADI). Next, the ASES score and SPADI of the affected side were compared with those of the unaffected side. We also investigated the outcomes of conservative treatments for midshaft clavicle fractures. While we defined patients whose ASES score on the affected side was lower and whose SPADI was higher than the unaffected side as the symptomatic group, other patients were classified as the asymptomatic group.

**Fig. 2:**
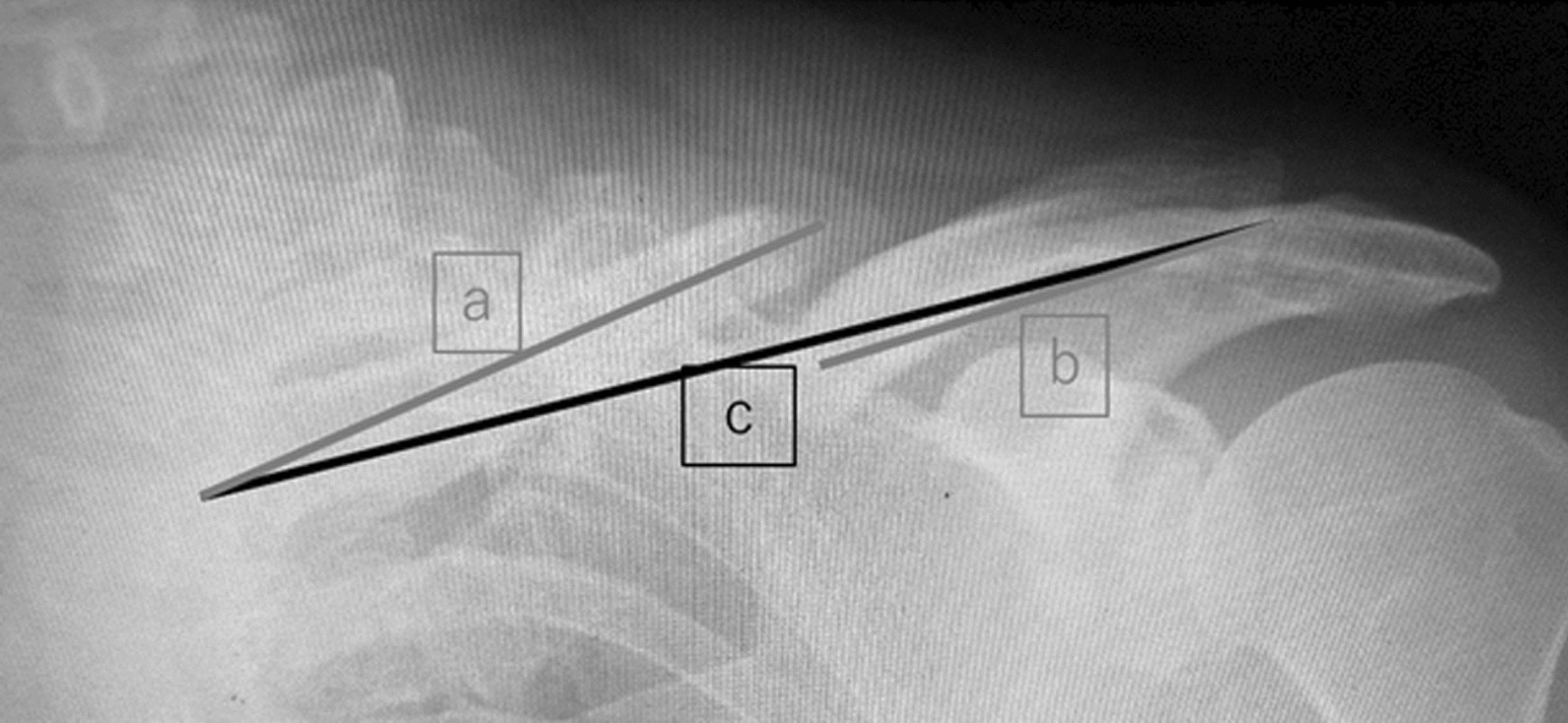
Differences between the sum of the fragments’ length and the direct distance from the proximal to the distal clavicular edge were calculated as the absolute value (|a + b − c|). The proportional clavicular length change was |a + b − c|/(a + b)

**Fig. 3:**
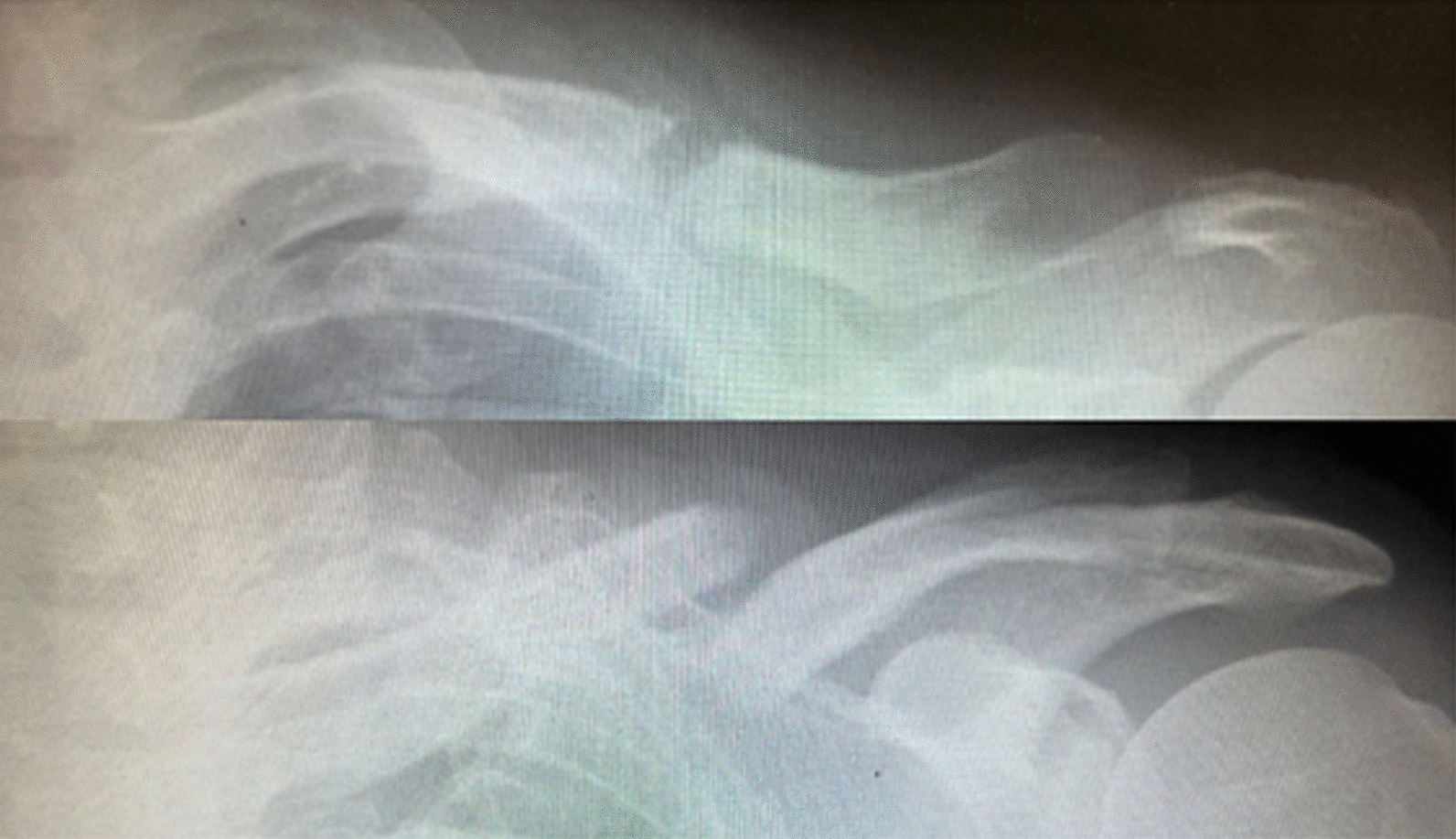
Upside is lengthened clavicle, downside is shortened clavicle

Subsequently, we calculated proportional changes in the clavicular length on a plain radiograph when the callus was formed. First, the sum of the fragments' length on the plain front radiograph was considered the clavicular length before trauma (Fig. 2 a + b). Then, the direct distance from the proximal clavicular edge to the distal clavicular edge of the affected side was considered the clavicular length after trauma (Fig. 2c). Afterward, clavicular length differences before and after the trauma were calculated as the absolute value (Fig. 2 |a + b − c|) because lengthened and shortened clavicles were observed (Fig. 3). Proportional changes in the clavicular length were calculated by dividing that absolute value by the clavicular length before trauma (Fig. 2 |a + b − c|/(a + b)). Additionally, we measured displacements on plain radiographs when the callus was formed, after which the longer displacement was chosen either on the plain front or the oblique clavicle radiograph. Furthermore, we assessed whether clavicles on the latest plain radiograph were nonunion. Then, the symptomatic group's proportional changes in clavicular length and displacement were compared with the asymptomatic group. Later, the rate of nonunion of lengthened and shortened cases was also assessed.

## Statistics

The Wilcoxon signed-rank test was used to compare the affected and unaffected sides of the ASES score and SPADI. Fisher's exact test compared the symptomatic and asymptomatic groups based on the understudied genders. Furthermore, the Mann–Whitney U test was used to compare the symptomatic and asymptomatic groups based on age, term after trauma, proportional clavicular length changes, and displacement. Finally, a *p*-value of < 0.05 was considered significant.

## Results

There were no complications such as paresthesia. Callus formations were observed on plain radiographs at 2.1 ± 0.6 months. Bone unions were achieved in 34 patients at 4.7 ± 3.3 months. Results showed that while the ASES scores of the affected side was 96.2 ± 8.8 points, that of the unaffected side was 99.2 ± 2.4 points. Therefore, ASES scores of the affected side were significantly lower than that of the unaffected side (*p* = 0.032). While the SPADI of the affected side was 15.0 ± 13.8 points, that of the unaffected side was 0.6 ± 1.8 points. Hence, the SPADI of the affected side was significantly higher than that of the unaffected side (*p* = 0.011). In the symptomatic group, the ASES score of the affected side was 84.0 ± 11.7 points whereas that of the unaffected side was 97.8 ± 2.8 points; the SPADI of the affected side was 21.0 ± 22.3 points and that of the unaffected side was 2.0 ± 3.2 points.

This research understudied nine patients (23.7%) in the symptomatic group, six males and three females. While their mean age during the survey was 64.1 ± 18.9 years, the mean term after trauma was 17.0 ± 3.8 years. In contrast, 29 patients (76.3%) were understudied in the asymptomatic group, 25 males and four females. While their mean age during the survey was 59.2 ± 15.2 years, the mean term after trauma was 17.0 ± 4.8 years. This study also observed no significant difference between the two groups' mean age in the survey period, terms after trauma, and gender. Furthermore, although the clavicular length of six patients (66.7%) was shortened in the symptomatic group, the clavicular length of three patients (33.3%) was lengthened. Alternatively, in the asymptomatic group, while clavicular lengths of 17 patients (58.6%) were shortened, clavicular lengths of three patients (10.3%) were lengthened, and clavicular lengths of nine patients (31%) remained unchanged. Results also showed that patients in the symptomatic group suffered mainly from reduced range of motion (ROM) of the shoulders and shoulder pain (Table 1). Moreover, five of the six patients who had a shortened clavicle in the symptomatic group had pains when placing their hands behind their neck, four of those six experienced pains when reaching on a high shelf and difficulty in washing their back. Two of the three patients in the symptomatic group who had a lengthened clavicle experienced difficulties when placing an object on a high shelf and throwing balls.

**Table 1:** Overview of patients’ characteristics in the symptomatic group

Patient	1	2	3	4	5	6	7	8	9
Age (years)	85	51	36	74	58	77	39	86	71
Gender	Female	Female	Male	Female	Male	Male	Male	Male	Male
Term since trauma (years)	17.5	12.4	13.3	18.8	12.7	15.6	20	20	23
Dominant side affected	No	No	Yes	Yes	Yes	Yes	No	No	No
Lengthening or shortening	Short	Short	Length	Length	Short	Short	Short	Length	Short
Change in clavicular length (%)	11.0	4.5	2.6	4.0	2.1	10.4	5.6	1.4	7.1
Displacement (mm)	20	11	9	7	20	14	6	10	12
Nonunion	+	−	−	−	−	−	−	−	−
ASES of affected side	56.6	85.0	91.6	86.6	83.3	84.9	94.0	96.0	78.0
ASES of unaffected side	93.3	100.0	95.0	96.6	100.0	95.0	100.0	100.0	100.0
SPADI of affected side	71.5	10.7	9.2	10.8	4.6	28.5	6.2	7.7	40.0
SPADI of unaffected side	9.2	0	4.6	3.1	0	0.8	0	0	0
Pain of shoulder	+	+	+	−	+	+	+	+	−
Narrow ROM	+	+	−	+	−	−	+	+	+

ASES: American Shoulder and Elbow Surgeons; SPADI: Shoulder Pain and Disability Index; ROM: Range of motion

**Fig. 4:**
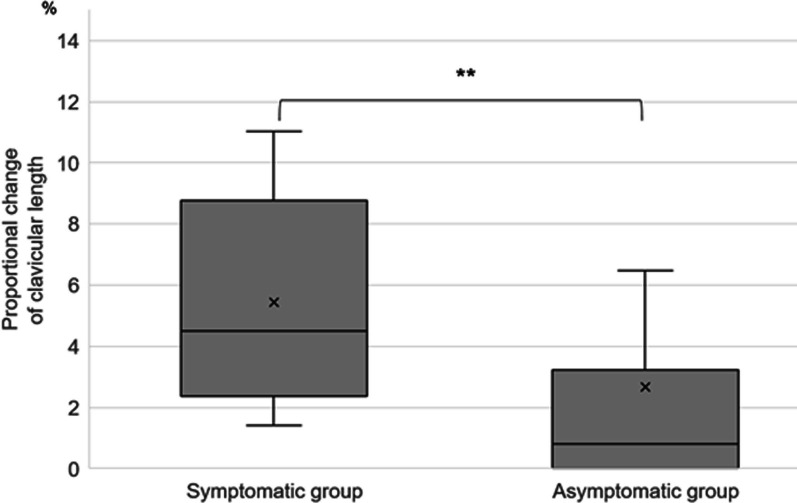
Comparison of the proportional clavicular length changes between the symptomatic and asymptomatic groups. Significance was set as *P* < 0.01 (denoted with the double asterisk)

**Fig. 5:**
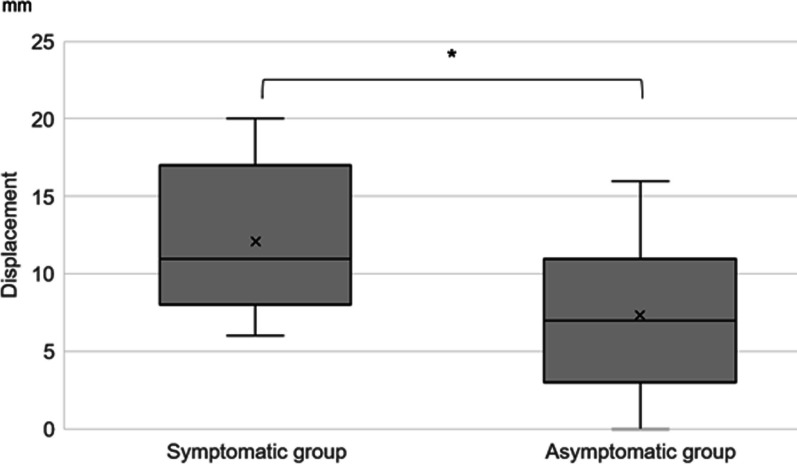
Comparison of the displacement between the symptomatic and asymptomatic groups. Significance was set as *P* < 0.05 (denoted with the asterisk)

The proportional clavicular length change in the symptomatic group was 5.4 ± 3.5%, and in the asymptomatic group was 2.7 ± 4.8%. As observed, the proportional change in the clavicular length of the symptomatic group was significantly larger than that in the asymptomatic group (*p* = 0.0078) (Fig. 4). Moreover, displacement was 12.1 ± 5.1 mm in the symptomatic group and 7.3 ± 4.7 mm in the asymptomatic group, with a significant difference between these groups (*p* = 0.028) (Fig. 5).

Investigations during 10.3 ± 5.7 months observed nonunion in four patients, one in the symptomatic group (11.1%) and three in the asymptomatic group (10.3%). Furthermore, six patients experienced clavicular lengthening; four males and two females were observed. Their mean age was 65.7 ± 17.1 years, and the mean term after trauma was 16.6 ± 2.9 years. However, 23 patients experienced clavicular shortening, 18 males and 5 females. Their mean age was 60.5 ± 17.3 years, and the mean term after trauma was 16.7 ± 4.9 years. Additionally, the proportional clavicular length change was 2.0 ± 1.2% in lengthened cases and 5.0 ± 5.3% in shortened cases. Specifically, three of the lengthened cases (50.0%) and one of the shortened cases (4.3%) had nonunion, with the three patients having nonunion in lengthened cases being all asymptomatic, whereas the other three in the lengthened cases who had union were symptomatic. One patient with nonunion in the shortened case had 11% shortening and a 20 mm displacement clavicle, which was a severe deformity.

## Discussion

We found that on conservative treatment of clavicle fractures, the affected side was more symptomatic than the unaffected side long term after trauma. Furthermore, patients tended to be symptomatic with larger proportional change in clavicular length and displacement. Many reports on the short-term outcome of conservative treatments for midshaft clavicle fracture have been discovered. In these studies, shortening did not result in short–term clinical changes after trauma [12, 13, 15]. Woltz et al. reported a systematic review showing that clavicle shortening did not relate to a shoulder function mean of 4.5 years after trauma [14]. Moreover, Figueiredo et al. reported that clavicle shortening did not affect the Disabilities of the Arm, Shoulder, and Hand (DASH) score during bone union even when shortening exceeded 2 cm at the point of trauma [12]. However, Su et al. reported that SPADI on the affected side was significantly worse than the unaffected side, with a mean of 33 months after trauma [18], and patients with a vertical displacement of more than 100% between the main fragments had unsatisfactory results in other reports [19, 20]. Additionally, in a study using cadaveric shoulders, Matsumura et al. reported that clavicle shortening of 10% or more impaired the scapula's external rotation and posterior tilting [21]. Therefore, the clavicle shortening, reducing external rotation and posterior tilting of the scapula is considered the cause of the symptoms. In our study, the symptomatic group had a significantly greater lengthening or shortening and displacement than the asymptomatic group, suggesting that patients with greater clavicle length changes and displacements showed symptoms. Significant symptoms of clavicular shortening in the symptomatic group were pains when reaching behind their neck and difficulties in washing their back, which can be due to impaired external rotation and posterior tilting.

Few reports also exist on long-term results. It has been reported that the Constant score was significantly lower in patients with shortening (15%) or displacements (more than 23 mm) at an average of 8.7 years after trauma [16]. Furthermore, the QUICKDASH score and muscle strength of the affected side were not different from those of the unaffected side, with a mean of 13.5 years after trauma when mean clavicle shortening was 10.9 mm [17]. This study showed that although the proportional clavicular length change was 5.4% and displacement was 13.5 mm in the symptomatic group, the ASES score and SPADI were significantly worse on the affected side than the unaffected side with a mean of 17.0 years after the trauma. No report whose mean of term after trauma was longer than our study discovered. These symptoms can appear as time goes on, even with smaller proportional clavicular length changes and displacements. Treatment without malunion is necessary for midshaft clavicle fractures in the long term.

Some reports also showed that the rate of nonunion was significantly higher in conservative treatments than surgical treatments [6–8], and the nonunion rate of midshaft clavicle fractures treated conservatively was 15–20% [22]. In our study, the nonunion rate was 10.5%, which was relatively low, certainly due to the thorough management. We retightened the figure-of-eight bandages twice or three times a week rigidly to prevent clavicular shortening based on our management protocol. However, some cases with clavicular lengthening were observed. Nevertheless, no report about clavicular lengthening has been found. Furthermore, this study's lengthened cases were symptomatic or nonunion. Patients who had clavicular lengthening in the symptomatic group mainly experienced difficulties when placing their hands on a high shelf and throwing balls, which was considered to be due to anatomical abnormalities. Therefore, clavicular lengthening as well as shortening can be associated with unsatisfactory outcomes. This is a new insight that warrants further research.

The limitations of this study are the lack of a control group, the retrospective study, the heterogeneity of the patients concerning age and the gender disparity of the patients. The sample of symptomatic cases was small because a number of patients were lost during the follow-up period, and the evaluation, based only on plain radiograph, may have been inaccurate. Hence, this study is not an objective indicator but a patient-based evaluation. There are some patient-based evaluations of the shoulder. Among these, the DASH score is commonly used. However, it is not only an assessment of the shoulder but that of the whole arm. Hence, SPADI has been proposed as the most responsive shoulder tool. The ASES score also has good applicability for research and good responsiveness in assessing symptoms and functions of the shoulder [23], accounting for the reason we applied the ASES score and SPADI.

## Conclusion

We discovered that the shoulder side, which had become a midshaft clavicle fracture, was more symptomatic than the unaffected side 10–30 years after the trauma when midshaft clavicle fractures were treated conservatively. Moreover, during fracture with larger shortening, lengthening, or displacement when callus was formed, several patients became symptomatic. Therefore, treatment without malunion is desirable. We also observed rigidly fastened patients with a figure-of-eight bandage and some patients with clavicular lengthening. However, they were symptomatic or had nonunion.

## Data Availability

The datasets generated and analyzed during the current study are not publicly available due to Japanese personal information protection law, but are available from the corresponding author on reasonable request.

## Abbreviations

ASES: American Shoulder and Elbow Surgeons
DASH: Disabilities of the Arm, Shoulder, and Hand
ROM: Range of motion
SPADI: Shoulder Pain and Disability Index
